# Secondary lymphedema of limbs and chikungunya fever

**DOI:** 10.1590/1677-5449.190015

**Published:** 2019-05-22

**Authors:** Marcos Arêas Marques, Ana Letícia de Matos Milhomens, Juliana de Miranda Vieira, Fabricius Rocha Cardoso, Henrique Jorge Guedes

**Affiliations:** 1 Universidade do Estado do Rio de Janeiro – UERJ, Rio de Janeiro, RJ, Brasil.; 2 Universidade Federal do Estado do Rio de Janeiro – UNIRIO, Rio de Janeiro, RJ, Brasil.; 3 Instituto Nacional de Câncer, Rio de Janeiro, RJ, Brasil.; 4 Escola Paulista de Medicina, Universidade Federal de São Paulo – UNIFESP, São Paulo, SP, Brasil.

**Keywords:** chikungunya virus, chikungunya fever, lymphedema, complications, chronic disease, arbovirus infections

## Abstract

Certain systemic viral infections can be related to development of vascular complications, such as deep venous thrombosis and lymphedema of lower and upper limbs. These links have been well-established in patients with human immunodeficiency virus (HIV), hepatitis C, or influenza. Recently introduced into the American continent (2013), chikungunya virus is an arbovirus transmitted by mosquitoes of the *Aedes* genus and is the etiologic agent of chikungunya fever (CF), but its relationship to these vascular complications has not yet been consolidated. However, the CF outbreak that occurred during 2015 and 2016 resulted in the first cases described in the medical literature of acute and chronic vascular complications secondary to infection by this arbovirus. In this report, we describe the case of a patient who developed lymphedema of upper and lower limbs after an episode of CF.

## INTRODUCTION

Chikungunya fever (CF) is caused by the chikungunya virus (CHIKV), an arbovirus of the *Alphavirus* genus that is transmitted to humans via bites by female mosquitoes of the *Aedes* genus, and was first described in the 1950s in central Africa and later in other African countries and in Asia.^1^ In 2007, CHIKV arrived in Europe, causing a CF outbreak in Italy, and it is believed that from this point onwards dissemination of CHIKV to new territories, such as Australia and the Western hemisphere began to occur.^1^^,^ 2 In December of 2013, the Pan American Health Organization published the first notification of autochthonous CHIKV transmission on the American continent.^3^ Clinically, CF is characterized by abrupt onset of high fever (> 38.9 °C), shivering, and photophobia, lasting approximately 7 days. In the majority of cases, patients also develop incapacitating polyathralgia, usually symmetrical, involving hips, elbows, fingers, toes, knees, and ankles, limiting locomotion for months or even years,^1^^-^ 3 in addition to maculopapular rash on the trunk, face, and extremities, itching, headaches, tiredness, nausea, vomiting, conjunctivitis, cervical lymphadenopathy, and myalgia.^1^^-^ 3 The most common laboratory findings during the acute phase of CF are leukopenia, thrombocytopenia, hypokalemia, and mild to moderate elevation of hepatic transaminases.^1^^,^ 2 The most common acute clinical complications of CF are secondary to involvement of the central nervous system, such as Guillain-Barré syndrome, and of the vision, including uveitis and retinitis.^2^ Chronically, development of syndromes affecting the joints is common, with long-lasting polyarthritis in 30 to 40% of cases.^1^^,^ 3 To date, vascular involvement has been described little in the medical literature in relation to CF and is normally restricted to Raynaud’s phenomenon persisting beyond the acute phase.^1^^,^ 2 However, since the CHIKV outbreaks in 2015 and 2016, other vascular manifestations, such as deep venous thrombosis and lymphedema, have also been occasionally described in the medical literature.^1^^,^ 4 ^,^ 5 Below, we describe the case of a patient who developed lymphedema of upper and lower limbs after a laboratory-confirmed CHIKV infection.

## CASE DESCRIPTION

**Figure 1:**
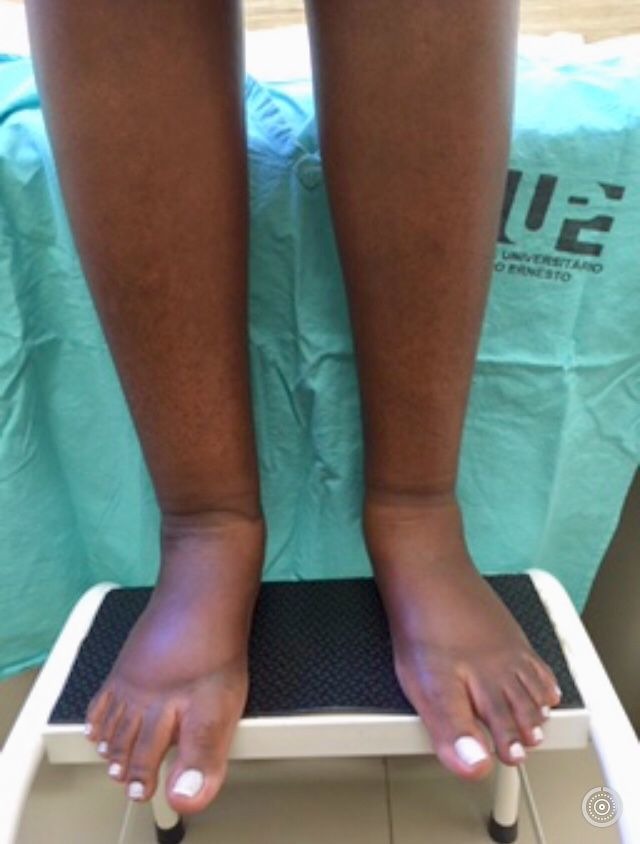
Lymphedema of lower limbs, more severe on the right.

**Figure 2:**
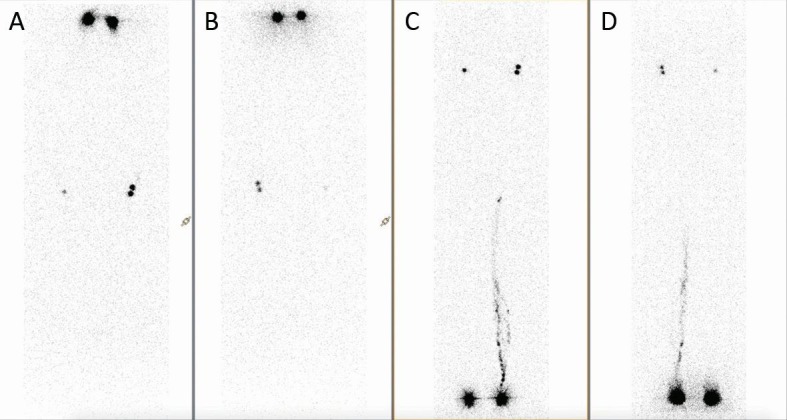
Semiquantitative lymphoscintigraphy of the upper limbs (A and B) and lower limbs (C and D). Images were captured 15, 30, 60, and 120 minutes after interdigital administration of 2.0 mCi 99m TC-Sn.

The patient was a 33-year-old, brown-skinned female, who sought care at a walk-in health center (HC) with a history of fever (> 39 °C), shivering, asthenia, nausea, vomiting, rash affecting the face and trunk, headaches, a stiff neck in the morning, and symmetrical polyarthritis of small and large joints (hips, shoulder, knees, elbows, fingers and toes). A blood test revealed leukopenia (2,190 leukocytes/µL) and elevated hematocrit (56%), probably caused by hemoconcentration secondary to dehydration. The patient was discharged from the HC with instructions to rest and take analgesics (paracetamol) and anti-inflammatories (naproxen), if needed for joint pain. Fifteen days later, she presented at the Rheumatology Service run by the Hospital Universitário Pedro Ernesto, Universidade do Estado do Rio de Janeiro (UERJ), complaining of exacerbation of the arthralgia and edema involving upper and lower limbs, despite regularly taking the medication prescribed, preventing her from working and practicing sport (muay thai). At this point, the patient no longer had fever or rash and was medicated with a combination of paracetamol-codeine and nimesulide. Around 7 days later, she returned to the Rheumatology Service, complaining of worsened edema in the upper limbs, especially on the right, and persistent arthralgia. At this consultation, laboratory tests were ordered to investigate her acute symmetrical polyarthritis and a hypothesis of CF was ventured and confirmed by reactive serology (Chikungunya antivirus ELISA IgG - Euroimmun®, > 22 UR/mL). Since the patient’s upper limb edema continued to worsen, she was referred to the Angiology Service at the Hospital Universitário Pedro Ernesto, Universidade do Estado do Rio de Janeiro (UERJ) for assessment. Physical examination found progressive edema involving all four limbs, with no improvement in response to rest, a positive Stemmer sign, and right upper and lower limbs with greater volume than the contralateral limbs (Figure 1). After discussing the case in a team meeting, color Doppler ultrasonography of the deep venous system of the upper and lower limbs was requested, to rule out deep venous thrombosis, and semiquantitative lymphoscintigraphy of the upper and lower limbs was performed to guide physiotherapy. Lymphoscintigraphy (Figure 2) demonstrated slow lymph flow in upper and lower limbs, and collateral lymph flow in the left leg and popliteal lymph node. The patient was referred to the Physiotherapy Service for complex physiotherapy.

## DISCUSSION

Viral infections can provoke acute and chronic systemic inflammatory processes that often involve the osteoarticular system, causing acute polyarthritis that can sometimes become chronic.^1^^-^ 4 In CF, approximately 48% of patients have chronic arthritis lasting at least 6 months.^3^ This has been confirmed in the laboratory by persistent elevation of certain inflammatory markers, such as interleukin-6, for example.^3^ While there are very few references in the medical literature on the association between CHIKV and acute and chronic vascular complications,^1^^,^ 4 ^,^ 5 the recent outbreak of CF on the American continent, in 2015, provoked a considerable increase in both incidence and prevalence of this arbovirus and, as a result, of its less common complications. The emergence of lymphedema involving lower and upper limbs in patients who have had CF probably has multifactorial origins. These patients may have a reduced lymphatic reserve, caused by anatomic and/or dynamic, congenital and/or acquired changes, with the result that the system is unable to deal with the demand caused by increase lymph production secondary to the articular inflammatory process characteristic of infection by CHIKV. Additionally, these patients very often have movement chronically limited by intense pain, or may even be practically restricted to bed, reducing muscle contractions in the limbs and reducing one of the physiological mechanisms of lymph drainage.^6^ In the case described, the patient practiced martial arts regularly (muay thai) and was employed as a hairdresser, but had to cease both activities because of incapacitating arthralgia. Restricted movement can also provoke increase in body mass index, which can be another factor in maintenance of lymphedema, since it can limit patients’ return to their daily activities even more.

## CONCLUSIONS

The vascular complications of CF began to be described sporadically after the outbreaks in 2015 and 2016. Thee pathophysiology of lymphedema secondary to infection by CHIKV has not yet been elucidated, but it clearly involves several different mechanisms which, in combination, can provoke or exacerbate lymphatic dysfunction and, therefore, induce a chronic condition.
