# Supplementary material for: Secondary lymphedema of limbs and chikungunya fever
Source: J Vasc Bras. 2019 May 22;18:e20190015. doi: 10.1590/1677-5449.190015 (PMC6634949; doi:10.1590/1677-5449.190015)
Supplement: Translation

# Translation: Linfedema secundário de membros e febre chicungunha

**Authors:** Marcos Arêas Marques, Ana Letícia de Matos Milhomens, Juliana de Miranda Vieira, Fabricius Rocha Cardoso, Henrique Jorge Guedes

**Affiliations:** 1 Universidade do Estado do Rio de Janeiro – UERJ, Rio de Janeiro, RJ, Brasil.; 2 Universidade Federal do Estado do Rio de Janeiro – UNIRIO, Rio de Janeiro, RJ, Brasil.; 3 Instituto Nacional de Câncer, Rio de Janeiro, RJ, Brasil.; 4 Escola Paulista de Medicina, Universidade Federal de São Paulo – UNIFESP, São Paulo, SP, Brasil.

**Keywords:** vírus chicungunha, febre de chicungunha, linfedema, complicações, doença crônica, infecções por arbovírus

## Abstract

Algumas infecções virais sistêmicas podem estar relacionadas ao desenvolvimento de complicações vasculares, como trombose venosa profunda e linfedema de membros inferiores e superiores. Essa relação já está bem estabelecida em pacientes portadores do vírus da imunodeficiência humana (HIV), hepatite C ou *influenza*. Recentemente introduzido no continente americano (2013), o vírus chicungunha, um arbovírus transmitido pelo mosquito do gênero *Aedes* e agente etiológico da febre chicungunha (FC), ainda não tem essa relação bem sedimentada. Porém, o surto de FC, ocorrido entre 2015 e 2016, fez com que fossem descritos na literatura médica os primeiros casos de complicações vasculares agudas e crônicas secundárias à infecção por essa arbovirose. Neste relato de caso, descrevemos uma paciente que desenvolveu linfedema de membros superiores e inferiores após quadro de FC.

## INTRODUÇÃO

A febre chicungunha (FC) é causada pelo vírus chicungunha (CHIKV), um arbovírus do gênero *Alphavirus*, transmitido aos humanos por meio da picada da fêmea de mosquito do gênero *Aedes*, inicialmente descrita nos anos 1950, na África central, e posteriormente em outros países africanos e na Ásia^1^. Em 2007, o CHIKV chegou ao continente europeu, causando um surto de FC na Itália, e acredita-se que a partir desse momento houve disseminação do CHIKV em direção a novos territórios, como a Austrália e o hemisfério oeste^1^^,^ 2. Em dezembro de 2013, a Organização Pan-Americana de Saúde emitiu, pela primeira vez, um alerta de transmissão autóctone de CHIKV no continente americano^3^. Clinicamente, a FC se caracteriza por início abrupto de febre alta (> 38,9 °C), calafrios e fotofobia, que duram aproximadamente 7 dias. Na maioria dos casos, os pacientes também apresentam poliartralgia incapacitante, usualmente simétrica, que acomete quadril, cotovelos, dedos, joelhos e tornozelos, limitando a locomoção do paciente por meses ou até mesmo anos^1^^-^ 3, além de exantema maculopapular em tronco, face e extremidades, prurido, cefaleia, fadiga, náuseas, vômitos, conjuntivite, linfadenopatia cervical e mialgia^1^^-^ 3. As alterações laboratoriais mais frequentes na fase aguda da FC são leucopenia, trombocitopenia, hipocalemia e elevação leve a moderada das transaminases hepáticas^1^^,^ 2. As complicações clínicas agudas mais comuns da FC são secundárias ao acometimento do sistema nervoso central, como a síndrome de Guillain-Barré, e do sistema ocular, como a uveíte e a retinite^2^. Cronicamente, é comum o desenvolvimento de síndromes articulares, com longa persistência de poliartrite em 30 a 40% dos casos^1^^,^ 3. O acometimento vascular na FC é ainda pouco descrito na literatura médica e está normalmente restrito ao fenômeno de Raynaud persistente após a sua fase aguda^1^^,^ 2. Porém, desde os surtos de CHIKV, nos anos de 2015 e 2016, outras manifestações vasculares, como trombose venosa profunda e linfedema, também têm sido pontualmente relatadas na literatura médica^1^^,^ 4 ^,^ 5. Abaixo, descrevemos o caso de uma paciente que desenvolveu linfedema de membros superiores e inferiores após infecção por CHIKV confirmada laboratorialmente.

## DESCRIÇÃO DO CASO

Paciente do sexo feminino, 33 anos de idade, parda, procurou o pronto-atendimento (PA) com história de febre (> 39 °C), calafrios, astenia, náuseas, vômitos, exantema de face e tronco, cefaleia, rigidez cervical matinal e poliartrite simétrica de pequenas e grandes articulações (quadril, ombro, joelhos, cotovelos, pododáctilos e quirodáctilos). Foi solicitado hemograma, que demonstrou leucopenia (2.190 leucócitos/µL) e aumento do hematócrito (56%) por provável hemoconcentração secundária à desidratação. A paciente recebeu alta do PA com orientação de repouso e uso de analgésico (paracetamol) e anti-inflamatório (naproxeno) em caso de dor articular. Quinze dias após, procurou o Serviço de Reumatologia do Hospital Universitário Pedro Ernesto da Universidade do Estado do Rio de Janeiro (UERJ), pois relatava piora da artralgia e do edema de membros superiores e inferiores, mesmo com o uso regular das medicações prescritas, incapacitando-a de exercer as suas atividades profissionais e esportivas (muay-thay). Nesse momento, a paciente já não apresentava mais febre e exantema, sendo medicada com a associação de paracetamol-codeína e nimesulida. Cerca de 7 dias após, retornou novamente ao Serviço de Reumatologia com queixa da piora de edema de membros superiores, especialmente à direita, e manutenção da artralgia. Nessa consulta, foram solicitados exames laboratoriais para investigação de poliartrite simétrica aguda, e foi levantada a hipótese de FC, confirmada por sorologia reagente (antivirus Chikungunya ELISA IgG - Euroimmun®, > 22 UR/mL). Como a paciente evoluiu com piora de edema dos membros superiores, foi solicitada uma avaliação pelo Serviço de Angiologia do Hospital Universitário Pedro Ernesto da Universidade do Estado do Rio de Janeiro (UERJ). Ao exame físico, a paciente apresentava edema progressivo nos quatro membros, que não melhorava com o repouso, com sinal de Stemmer positivo e mais volumoso em membros superior e inferior direitos (Figura 1). Após discussão do caso em equipe, optou-se por solicitar eco-Doppler colorido venoso profundo de membros superiores e inferiores, para descartar trombose venosa profunda, e linfocintilografia semiquantitativa de membros superiores e inferiores para orientar o tratamento fisioterapêutico. A linfocintilografia (Figura 2) demonstrou lentificação do fluxo linfático em membros superiores e inferiores, além de colateralização de fluxo linfático em perna e linfonodo poplíteo à esquerda. A paciente foi encaminhada ao Serviço de Fisioterapia para realização de terapia física complexa.

**Figura 1:** Linfedema de membros inferiores, predominando à direita.

**Figura 2:** Linfocintilografia semiquantitativa de membros superiores (A e B) e inferiores (C e D). Foram realizadas imagem 15, 30, 60 e 120 minutos após a administração interdigital de 2,0 mCi 99m TC-Sn.

## DISCUSSÃO

Infecções virais podem provocar processos inflamatórios sistêmicos agudos e crônicos, que acometem especialmente o sistema osteoarticular, levando a um quadro de poliartrite aguda que, eventualmente, pode cronificar^1^^-^ 4. Na FC, aproximadamente 48% dos pacientes mantêm um quadro de artrite crônica por pelo menos 6 meses^3^. Esse fato é corroborado laboratorialmente pela elevação persistente de alguns marcadores inflamatórios, como a interleucina-6, por exemplo^3^. Embora haja pouquíssimas referências na literatura médica sobre a associação entre o CHIKV e complicações vasculares agudas e crônicas^1^^,^ 4 ^,^ 5, o recente surto de FC no continente americano em 2015 provocou um aumento importante na incidência e na prevalência dessa arbovirose, e portanto, de suas complicações menos frequentes. O aparecimento de linfedema de membros inferiores e superiores nos pacientes acometidos por FC provavelmente tem origem multifatorial. Esses pacientes podem ter uma reserva linfática diminuída, por alterações anatômicas e/ou dinâmicas, congênitas e/ou adquiridas, e, portanto, não conseguem suprir a demanda causada pelo aumento da produção de linfa secundário a um processo inflamatório articular característico da infecção pelo CHIKV. Além disto, esses pacientes ficam muitas vezes com a sua movimentação cronicamente limitada pela dor intensa, ou até mesmo praticamente restritos ao leito, o que diminuiria a contração muscular dos membros, acarretando assim a diminuição de um dos mecanismos fisiológicos da drenagem linfática^6^. No caso relatado, a paciente era praticante regular de artes marciais (muay-thay) e trabalhava como cabeleireira, e teve que abandonar ambas as atividades pela artralgia incapacitante. A restrição da movimentação também pode provocar o aumento do índice de massa corporal, o que pode ser um adjuvante da manutenção do linfedema, por limitar mais ainda os pacientes e dificultar o retorno às suas atividades usuais.

## CONCLUSÕES

As complicações vasculares da FC começaram a ser pontualmente descritas a partir do surto ocorrido nos anos de 2015 e 2016. A fisiopatologia do linfedema secundário a infecção pelo CHIKV ainda não está esclarecida, mas certamente envolve diversos mecanismos que, associados, podem provocar ou potencializar uma disfunção linfática e, portanto, levar a um quadro crônico.
